# Impact of Triclosan on Female Reproduction through Reducing Thyroid Hormones to Suppress Hypothalamic Kisspeptin Neurons in Mice

**DOI:** 10.3389/fnmol.2018.00006

**Published:** 2018-01-19

**Authors:** Xin-Yuan Cao, Xu Hua, Jian-Wei Xiong, Wen-Ting Zhu, Jun Zhang, Ling Chen

**Affiliations:** ^1^State Key Lab of Reproductive Medicine, Nanjing Medical University, Nanjing, China; ^2^Department of Physiology, Nanjing Medical University, Nanjing, China; ^3^MOE and Shanghai Key Laboratory of Children’s Environment Health, Xinhua Hospital, Shanghai Jiao Tong University School of Medicine, Shanghai, China

**Keywords:** triclosan, estrous cycle, kisspeptin, thyroid hormones, prolactin

## Abstract

Triclosan (TCS), a broad-spectrum antimicrobial agent, is widely used in clinical settings and various personal care products. The aim of this study was to evaluate the influence of TCS on reproductive endocrine and function. Here, we show that the exposure of adult female mice to 10 or 100 mg/kg/day TCS caused prolongation of diestrus, and decreases in antral follicles and corpora lutea within 2 weeks. TCS mice showed decreases in the levels of serum luteinizing hormone (LH), follicle-stimulating hormone (FSH) and progesterone, and gonadotrophin-releasing hormone (*GnRH*) mRNA with the lack of LH surge and elevation of prolactin (PRL). TCS mice had lower kisspeptin immunoreactivity and *kiss1* mRNA in anteroventral periventricular nucleus (AVPV) and arcuate nucleus (ARC). Moreover, the estrogen (E2)-enhanced AVPV-kisspeptin expression was reduced in TCS mice. In addition, the serum thyroid hormones (triiodothyronine (T3) and thyroxine (T4)) in TCS mice were reduced with increases in levels of thyroid stimulating hormone (TSH) and thyroid releasing hormone (TRH). In TCS mice, the treatment with Levothyroxine (L-T4) corrected the increases in PRL, TSH and TRH; the administration of L-T4 or type-2 dopamine receptors agonist quinpirole inhibiting PRL release could rescue the decline of kisspeptin expression in AVPV and ARC; the treatment with L-T4, quinpirole or the GPR45 agonist kisspeptin-10 recovered the levels of serum LH and FSH and progesterone, and *GnRH* mRNA. Furthermore, TCS mice treated with L-T4 or quinpirole resumed regular estrous cycling, follicular development and ovulation. Together, these results indicate that exposing adult female mice to TCS (≥10 mg/kg) reduces thyroid hormones causing hyperprolactinemia that then suppresses hypothalamic kisspeptin expression, leading to deficits in reproductive endocrine and function.

## Introduction

Triclosan (2,4,4′-trichloro-2′-hydroxy-diphenyl ether, TCS) is a synthetic antibacterial compound largely utilized in personal and household products such as toothpastes, shampoos, cosmetics, antibacterial soaps, deodorants, kitchen utensils, bedding and clothing (Rodricks et al., 2010). Given the ubiquity of such products, humans are continually exposed to TCS (Chalew and Halden, 2009; Reiss et al., 2009) through both skin and oral absorption pathways (Moss et al., 2000; Sandborgh-Englund et al., 2006). TCS was recently detected in 100% and 51% of urine and cord blood samples, respectively, obtained from 181 expectant mothers in New York (Pycke et al., 2014).

TCS exhibits several additional biological activities that are unrelated to its antibacterial action, many of which affect specifically the endocrine system. Reports of the estrogenic activity of TCS are mixed, for example it can amplify estrogen action *in vivo* (Jung et al., 2012; Louis et al., 2013), or reduces sulfonation of estradiol and estrone (James et al., 2010), but acts as an antagonist at the estrogen receptor (ER; Ahn et al., 2008). A large body of evidence indicates that the exposure of female rats to TCS reduces the thyroid hormones (Stoker et al., 2010). The oral administration of TCS decreases dose-dependently the level of circulating thyroxine (T4) in weanling female rats (Witorsch, 2014). The treatment with TCS in pregnant rats decreases total serum triiodothyronine (T3) and T4 (Rodríguez and Sanchez, 2010). This decrease in thyroid hormone results in reduced negative feedback on the hypothalamus-pituitary axis enhances thyroid releasing hormone (TRH) secretion, which would in turn promote excess prolactin (PRL) secretion (Tohei et al., 2000). PRL elevation was found in 21% of patients with overt hypothyroidism, and 8% of patients with subclinical hypothyroidism (Goel et al., 2015). Hyperprolactinemia is a major neuroendocrine-related cause of reproductive disturbances in women.

In females, the estrous cycle and ovarian function are controlled by the hypothalamic-pituitary-gonadal (HPG) axis. The pulse release of gonadotrophin-releasing hormone (GnRH)/luteinizing hormone (LH) and generation of preovulatory surge-like LH release (LH-surge) are altered by the feedback action of estradiol (E2; Adachi et al., 2007). Kisspeptin neurons in the arcuate nucleus (ARC) and anteroventral periventricular nucleus (AVPV) have been demonstrated to be responsible for mediating the feedback effects of E2 on GnRH/LH secretion (Kinoshita et al., 2005). Approximately 90% of GnRH neurons express the kisspeptin receptor G protein-coupled receptor 54 (GPR54; Pinilla et al., 2012). The activation of GPR54 can increase the frequency and amount of the GnRH/LH secretion (Stathatos et al., 2005). Growing evidence indicates that, through GnRH, ARC-kisspeptin neurons control tonic pulsatile LH release (Qiu et al., 2016; Clarkson et al., 2017), and AVPV-kisspeptin neurons regulate the generation of the LH surge to induce ovulation (Ohkura et al., 2009). A high proportion of ARC- and AVPV-kisspeptin neurons in female rats also express PRL receptors (Kokay et al., 2011). Several lines of evidence suggest that high PRL levels inhibit ARC-kisspeptin expression during lactation (Araujo-Lopes et al., 2014). Exogenous PRL administration prevents the occurrence of preovulatory LH surges in intact female rats (Araujo-Lopes et al., 2014). Therefore, investigating whether TCS through decreasing thyroid hormones to increase PRL secretion affects the hypothalamic kisspeptin neurons is of great interest to us.

To evaluate influence of TCS on reproductive endocrine and underlying molecular mechanisms, we in the present study examined the estrous cycle and ovarian morphology, hypothalamic kisspeptin expression, hypothalamic-pituitary-reproductive endocrine, activities of hypothalamic-pituitary-thyroid axis and serum PRL concentration in adult female mice treated with TCS (1, 10, or 100 mg/kg) for 50 days. Our results suggest that in adult female mice, TCS exposure (≥10 mg/kg) through reducing thyroid hormones causes hyperprolactinemia that then suppresses hypothalamic kisspeptin synthesis, thereby disrupting the reproductive endocrine and ovarian function.

## Materials and Methods

### Animals

This study was carried out in accordance with the recommendations of “experimental animals guidelines established by the Laboratory Animal Research Institute”. The protocol was approved by “Ethical Committee of the Nanjing Medical University”. Female ICR mice (Oriental Bio Service Inc., Nanjing) at 12 weeks of age (30 ± 2 g) were housed in stainless steel cages with wood bedding to minimize additional exposure to endocrine disrupting chemicals (temperature 23 ± 2°C, humidity 55 ± 5%, 12:12 h light/dark cycle, and lights from 06:00) in Animal Research Center of Nanjing Medical University. They received food and water *ad libitum*. Their body weight was measured every day. All efforts were made to minimize animal suffering. Every early morning (09:00 h), estrous cyclicity was examined using vaginal cytology (Caligioni, 2009).

### Administration of TCS

TCS (>99% purity) was purchased from Sigma-Aldrich (Sigma-Aldrich Inc., St. Louis, MO, USA). TCS was dissolved in dimethyl sulfoxide (DMSO), and then diluted with corn oil (final concentration of 0.5% DMSO). After 3–4 regular estrous cycles were determined, the mice were given the oral intake of TCS at doses of 1, 10 and 100 mg/kg per day at 08:00 h. A recent study (Wang et al., 2015) reported that the urinary TCS level in gestational mice receiving 10 mg/kg TCS is equivalent to high urinary TCS levels of spontaneous abortion patients. Thus, these doses resemble the exposure level to TCS in spontaneous abortion patients. Control mice were treated with oral intake of 0.5% DMSO.

### Measurement of TCS

To measure urinary TCS levels, each mouse was housed in a metabolic cage for 5 days. The urine samples (0.2–0.3 ml/mouse) within 12 h after the administration of TCS were collected and stored at −80°C until measurement. The TCS concentrations of urinary (1 ml/mouse) were measured using an established method (Wang et al., 2017). Briefly, the urine samples hydrolyzed with β-glucuronides (Type H-1 from Helix Pomatia, Sigma-Aldrich, St. Louis, MO, USA) were concentrated by a solid phase extraction (SPE; 500 mg/3 mL; Supelco, ENVI-18) and analyzed using liquid chromatography electrospray ionization tandem mass spectrometry (HPLC-MS/MS, Agilent 1290-6490, Agilent Technologies, Santa Clara, CA, USA). Analysts were blinded to all information concerning subjects during the tests.

### Histological Examination of Ovarian

The mice at diestrus were anesthetized with intraperitoneal (i.p.) injection chloral hydrate (400 mg/kg). Both ovaries were dissected and fixed in Bouin’s fluid. The samples were dehydrated through a graded series of alcohol, cleared in xylene, and then embedded in paraffin wax. After the sections (5 μm) were deparaffined and rehydrated, the sections were stained with hematoxylin and eosin (HE). The classification of follicular stages was made following the morphological criteria as described previously (Myers et al., 2004). Follicles were counted using a conventional light microscope (Olympus DP70, Japan) with a 40× objective. Antral follicles (early antral, antral and preovulatory follicles) and corpora lutea were counted in every 6th section (30 μm apart). Then, the numbers of antral follicles and corpora lutea were multiplied by 6 to give a total number in each ovary.

### Immunohistochemistry of Kisspeptin Neurons

Mice were anesthetized with chloral hydrate (400 mg/kg, i.p.) and perfused transcardially with 4% paraformaldehyde. Brains were transferred gradually into 15% and 30% sucrose until they settled. Sections (40 μm thick) through the AVPV (Bregma +0.50 to +0.02 mm) the ARC area (Bregma −1.46 to −1.70 mm; Marraudino et al., 2017) were cut using a cryostat. Free-floating sections were incubated in 0.5% sodium metaperiodate for 20 min and then in 1% sodium borohydride for 20 min. The sections were pre-incubated with 1% normal fetal goat serum for 60 min, and then incubated in rabbit anti-kisspeptin polyclonal antibody (1:1000, Catalog# AB9754, Millipore, Billerica, MA, USA) at 4°C for 24 h. Then, the sections were treated with biotin-conjugated goat anti-rabbit IgG (1:400; vector laboratories, Burlingame, CA, USA) at 37°C for 2 h. The immune-reactivity was visualized with the standard avidin-biotin complex reaction with Ni-3, 3-diaminobenzidine (DAB, Vector Laboratories). In every experiment, incubation of sections without the primary antibody served as negative controls for immunohisto-chemistry. Kisspeptin-positive (kisspeptin^+^) cells in AVPV (AVPV-kisspeptin^+^ cells) and ARC (ARC-kisspeptin^+^ cells) were observed by conventional light microscope (Olympus DP70; Olympus, Tokyo, Japan) with a 40× objective.

### Measurement of Serum Hormones

Orbital blood (~300 μl) was obtained under anesthetized conditions with chloral hydrate (400 mg/kg, i.p.) at 1600–1700 h. Serum (~100 μl) was separated by centrifugation at 4°C and stored at −80°C until assay. The levels of serum estradiol (E2), progesterone (P4), LH and follicle-stimulating hormone (FSH) were measured on the day of diestrus (*group I*). The measurement of T3, T4, thyroid stimulating hormone (TSH), thyrotropin-releasing hormone (TRH) and PRL had no limit for any estrous cyclicity (*group II*). The serum sample (5 μl) was needed for each assay (E2, P4, LH, FSH, PRL, T3, T4, TSH or TRH) using commercial enzyme-linked immunosorbent assay (ELISA) kits (Uscn Life Science Inc., Houston, TX, USA). The measurement of each sample was repeated 2 times to obtain an average value. The sensitivities were 47.1 pg/ml for T3, 1.4 ng/ml for T4, 19.3 pg/ml for TSH, 0.18 μIU/ml for TRH, 2.0 pg/ml for E2, 0.2 ng/ml for P4, 0.2 ng/ml for LH, 0.4 ng/ml for FSH and 0.4 ng/ml for PRL, respectively. The intra- and inter-assay coefficients of variation were 4.5% and 7.2% for T3, 4.3% and 7.5% for T4, 3.2% and 9.5% for TSH, 5.6% and 7.2% for TRH, 6.0% and 5.8% for E2, 5.8% and 8.4% for P4, 5.5% and 8.9% for LH, 4.3% and 10.3% for FSH, 4.7% and 4.9% for PRL. For determination of the LH surge, the repetitive blood sampling was undertaken at 1600, 1700 and 1800 h, respectively, on the day of proestrus (*group III*). The mice were anesthetized with ketamine (80 mg/kg) and xylazine (4 mg/kg) and a needle was inserted into a caudal vein at 1400 h. The mice were gently restrained in a cardboard tube, and the blood sample (~50 μl per time) was collected without anesthesia using heparinized syringes. After each blood collection, an equivalent volume of heparinized saline (5 U/ml normal saline; CP Pharmaceuticals Ltd, Wrexham, UK) was injected. Serum (~15 μl) was stored at −80°C for subsequent ELISA of LH.

### Reverse Transcription, Quantitative Polymerase Chain Reaction (RT-qPCR)

The POA area (0.76 mm anterior to Bregma and 0.50 mm posterior to Bregma; Mayer and Boehm, 2011), the AVPV area (0.50 mm anterior to Bregma and 0.02 mm posterior to Bregma) at proestrus and the ARC area (−1.46 mm anterior to Bregma and −1.70 mm posterior to Bregma; Marraudino et al., 2017) at diestrus were collected from the frozen slices (200 μm thick) of brain using 16-gauge stainless steel tubing, and then stored at −80°C until assay. Total RNA of POA, AVPV or ARC regions was isolated using Trizol reagent (Invitrogen, Camarillo, CA). RNA (1 μg) was reverse-transcribed into cDNA using a Prime-Script RT reagent kit (Takara) for quantitative PCR (ABI Step One Plus) in the presence of a fluorescent dye (SYBR Green I; Takara). The synthesized cDNA was stored at −20°C until qRT-PCR was performed. The following primers were used for real-time PCR as described previously (Xi et al., 2011): *GnRH* F-GGGAAAGAGAAACACTGAACAC, R-GGACAGTACATTCGAAGTGCT;* kiss1* F-GAATGATCTCAATGGCTTCTTGG, R-TTTCCCAGGCATTAACGAGTT; *GAPDH* F-ACCACAGTCCATGCCATCAC, R-TCCACCACCCTGTTGCTGTA. All samples were run in triplicate for each gene and for GAPDH (as housekeeping gene). There was no difference in GAPDH expression among the groups. The relative expression of genes was determined using the 2^−∆∆ct^ method with normalization to *GAPDH* expression. On the basis of melting curve analyses, there were no primer dimers or secondary products formed.

### Administration of Drugs

Levothyroxine (L-T4; Sigma-Aldrich Inc., St. Louis, MO, USA) dissolved in 0.9% saline was subcutaneously (s.c.) injected at dose of 20 μg/kg/day (Cao et al., 2017). Quinpirole (Quin; Sigma-Aldrich Inc., St. Louis, MO, USA) was dissolved in 0.9% saline and injected (i.p.) at dose of 2 mg/kg (Zhang et al., 2016).

Kisspeptin-10 [Kp-10, KiSS-1 (112–121)/metastin (45–54; human)] (Sigma-Aldrich Corp) was dissolved in DMSO, and then was diluted by 0.9% saline to a final concentration of 0.5% DMSO. For repeated intracerebroventricular (i.c.v.) injection of kisspeptin-10, the mice were anesthetized with chloral hydrate (400 mg/kg, i.p.) and then placed into a stereotaxic instrument (Stoelting, Wood Dale, IL, USA). A small hole (2 mm diameter) was drilled in the skull using a dental drill. A guide cannula (26-gauge, Plastics One, Roanoke, VA, USA) was implanted into the right lateral ventricle (0.3 mm posterior, 1.0 mm lateral and 2.5 mm ventral to Bregma) and anchored to the skull with three stainless steel screws and dental cement. On day 3 after surgery, the dummy cannula was removed from the guide cannula, and replaced by infusion cannulas (30 gauge) connected by polyethylene tubing (PE10; Becton Dickinson, Sparks, MD, USA) with a stepper-motorized micro-syringe (Stoelting, Wood Dale, IL, USA). The kisspeptin-10 (1 nmol/3 μl; Gottsch et al., 2004) was injected daily for successive 7 days. The injection of kisspeptin-10 was given at 30 min after TCS administration. This dose was selected on the basis of the previous report that kisspeptin-10 potently elicits the LH secretion (Navarro et al., 2004). The mice treated with injection (i.c.v.) of vehicle (0.9% saline) were served as the control group.

Mice were ovariectomized (OVX) under the anesthetized conditions with intramuscular injections of ketamine (80 mg/kg) and xylazine (4 mg/kg) at day 43 of TCS exposure. After surgery, the OVX mice received a subcutaneous implant of a silastic tubing (1.57 mm inside diameter; 3.18 mm outside diameter; 10 mm in length; Dow Corning, Midland, MI, USA) that was filled with 20 μg/ml of E2 in olive oil. The E2-treatment for 5 days produced a physiological level of serum E2 (20.71 ± 6.25 pg/ml) in adult female mice. On day 6 after implanting silastic tubing, the mice were given the injection (s.c.) of E2 at doses of 100 μg/kg for two consecutive days to produce a preovulatory high level of E2 (314.38 ± 66.91 pg/ml) that could exert a positive feedback action in AVPV-kisspeptin neurons (Wang et al., 2014). The silastic tubing filled with vehicle was implanted in OVX mice as the control group.

### Data Analysis/Statistics

**Figure 1:**
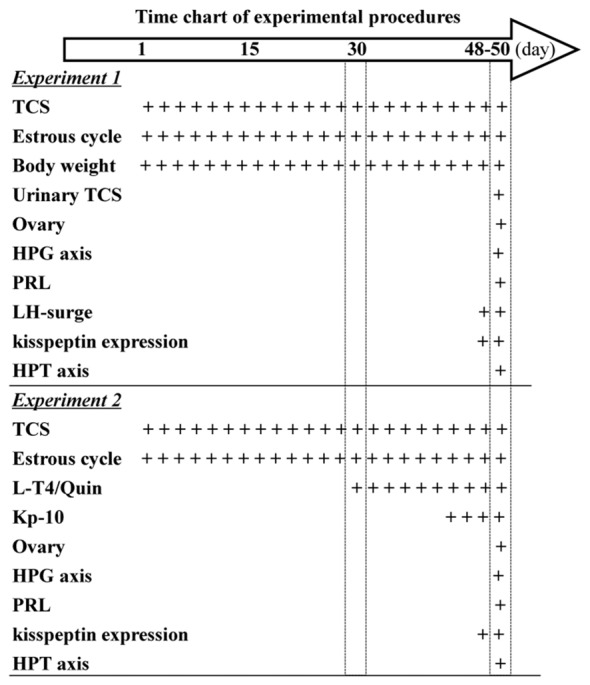
Time chart of experimental procedure. Hollow arrow indicates experimental time (day). “+” indicates the time of drugs administration and examination.

**Figure 2:**
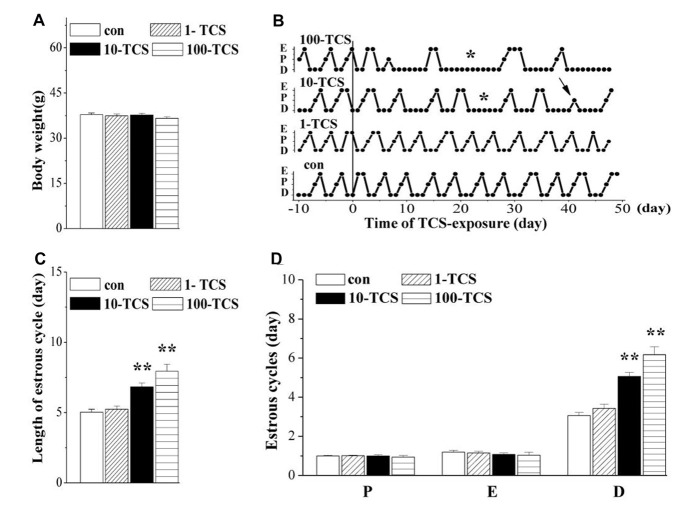
Triclosan (TCS) causes prolongation of diestrus. **(A)** Body weights (g) of mice. **(B)** Representative estrous cycles before and after TCS-exposure for 50 days. Connected dots indicate the time of diestrus (D), proestrus (P) or estrus (E), respectively. *: persistent diestrus; ↑: loss of proestrus. **(C)** Bar graph shows the mean time (day) of one estrous cycle within TCS-exposure. **P < 0.01 vs. control mice (one-way ANOVA). **(D)** Bars represent the mean length (day) of diestrus (D), proestrus (P), or estrus (E), respectively, per estrous cycle. **P < 0.01 vs. control mice (one-way ANOVA).

**Figure 3:**
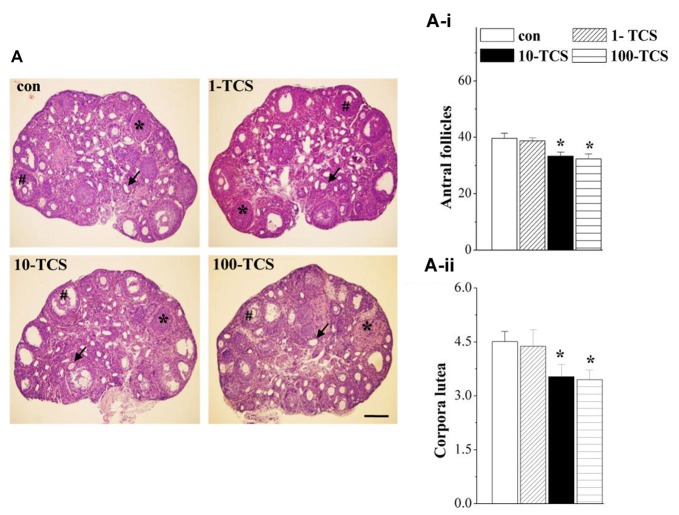
TCS affects follicle development and ovulation. **(A)** Representative images of ovaries stained with hematoxylin and eosin (HE) in control mice and TCS mice. #: antral follicles; ↑: atretic follicles; *: corpora luteum. Scale bars = 200 μm. **(A-i,A-ii)** Bar graphs represent the mean number of antral follicles and corpora luteum at diestrus, respectively. *P < 0.05 vs. control mice (one-way ANOVA).

**Figure 4:**
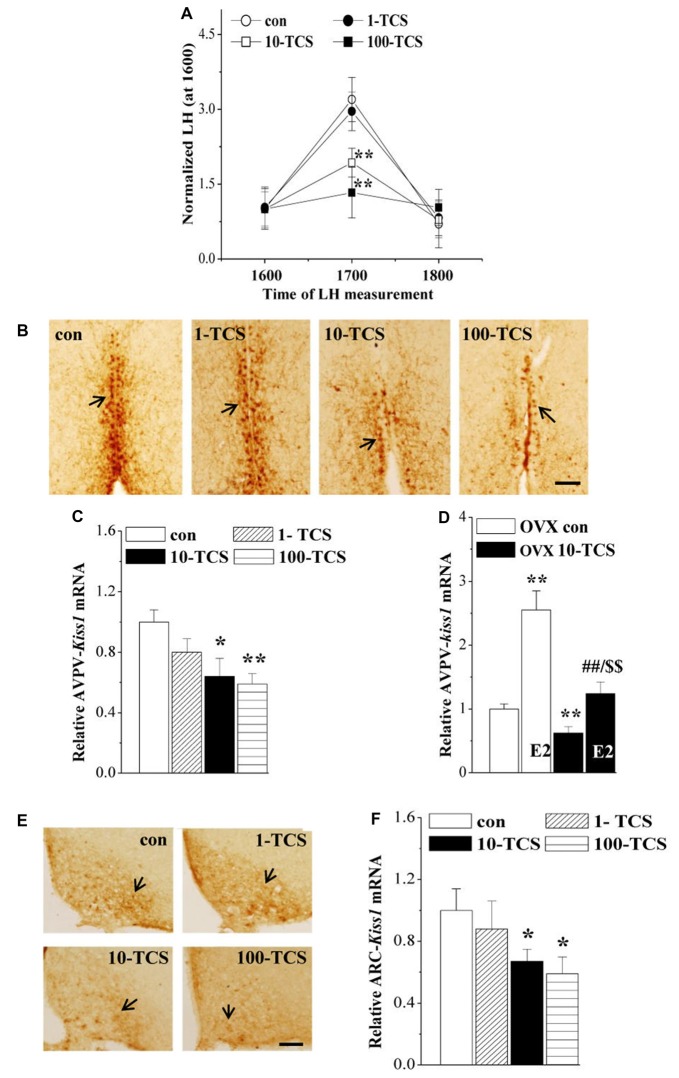
TCS suppresses luteinizing hormone (LH)-surge and kisspeptin expression. **(A)** Each point represents mean levels of LH that are normalized by the LH level at 1600. **P < 0.01 vs. control mice (one-way ANOVA). **(B)** Representative picture of kisspeptin immune-staining in anteroventral periventricularnucleus (AVPV) of proestrus control mice and TCS mice. Black arrows indicate AVPV-kisspeptin+ cells. Scale bars = 100 μm. **(C,D)** Bar graphs indicate the levels of AVPV-*kiss1* mRNA in proestrus mice or E2-treated OVX mice. *P < 0.05 and **P < 0.01 vs. control mice; ^##^*P* < 0.01 vs. OVX 10-TCS mice; ^$$^*P* < 0.01 vs. E2-treated ovariectomized (OVX) control mice (two-way ANOVA). **(E)** Representative picture of kisspeptin immune-staining in arcuate nucleus (ARC) of diestrus control mice and TCS mice. Scale bars = 100 μm. **(F)** Bars indicate the levels of ARC-*kiss1* mRNA in diestrus control mice and TCS mice. *P < 0.05 vs. control mice (one-way ANOVA).

**Figure 5:**
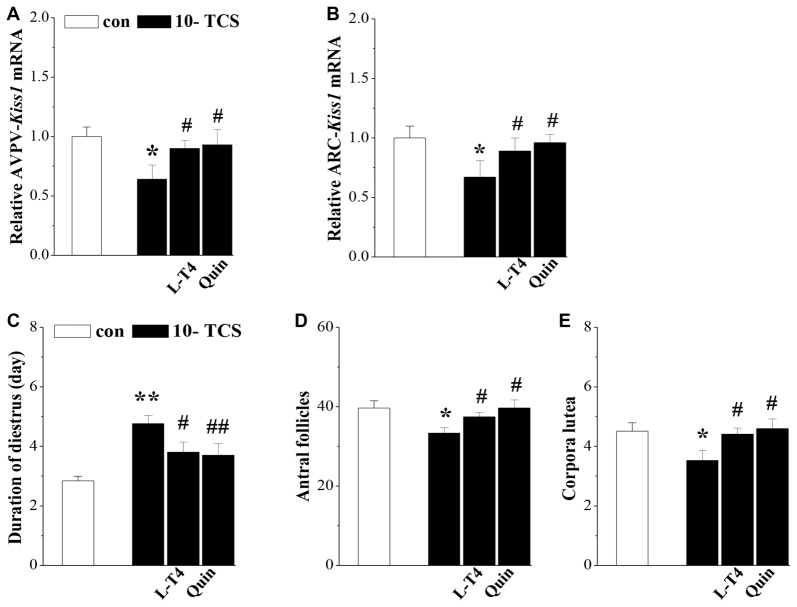
TCS-reduced kisspeptin causes persistent diestrus and ovary dysfunction. **(A,B)** Bar graphs indicate the levels of AVPV-*kiss1* mRNA and ARC-*kiss1* mRNA in 10-TCS mice treated with Levothyroxine (L-T4) or quinpirole (Quin). *P < 0.05 vs. control mice; ^#^*P* < 0.05 vs. 10-TCS mice (two-way ANOVA). Bar graphs represent the length (day) of diestrus **(C)**, the number of antral follicles **(D)** and corpora luteum at diestrus **(E)** in 10-TCS mice treated with L-T4 or Quin. *P < 0.05 vs. control mice; ^#^*P* < 0.05 vs. 10-TCS mice; **P< 0.01 vs. control mice; ^##^*P* < 0.01 vs. 10-TCS mice (two-way ANOVA).

All group data in the Figures 1–5 are expressed as the mean ± standard error of the mean (SEM); all group data in Tables 1, 2 are expressed as the mean ± standard deviation (SD). All statistical analyses were performed using SPSS software, version 16.0 (SPSS Inc., Chicago, IL, USA). When analyzing one-variable experiments with more than two groups, differences among means were analyzed using one-factor analysis of variance (ANOVA) followed by Bonferroni *post hoc* tests. Differences at *P* < 0.05 were considered statistically significant.

**Table 1:** Prolactin (PRL) and hypothalamic-pituitary-gonad and -thyroid hormones.

	Control	1-TCS	10-TCS	100-TCS
FSH (IU/L)	1.80 ± 0.42	1.79 ± 0.60	1.46 ± 0.25*	1.25 ± 0.49*
LH (IU/L)	1.08 ± 0.31	0.94 ± 0.38	0.80 ± 0.17*	0.71 ± 0.26**
E2 (pg/ml)	20.01 ± 3.88	17.29 ± 2.19	16.57 ± 4.40	15.74 ± 5.15
P4 (ng/ml)	4.27 ± 0.86	3.87 ± 0.66	3.52 ± 0.45*	3.18 ± 0.89*
PRL (ng/L)	54.95 ± 9.46	55.83 ± 7.25	61.34 ± 8.76*	62.04 ± 10.00*
*GnRH m*RNA	1.00 ± 0.13	0.95 ± 0.18	0.71 ± 0.21**	0.65 ± 0.19**
T4 (ng/ml)	54.63 ± 13.64	53.74 ± 12.72	44.65 ± 11.79*	26.19 ± 20.65**
T3 (ng/ml)	1.67 ± 0.35	1.62 ± 0.30	1.40 ± 0.36*	1.23 ± 0.55**
TSH (μIU/ml)	1.26 ± 0.23	1.21 ± 0.28	1.48 ± 0.43*	1.49 ± 0.35*
TRH (ng/L)	0.98 ± 0.30	1.05 ± 0.39	1.29 ± 0.47*	1.33 ± 0.50*

*P < 0.05, **P < 0.01 vs. control.

**Table 2:** Hypothalamic-pituitary trophic hormones and PRL.

	Control	10-TCS	10-TCS/L-T4	10-TCS/Quin	10-TCS/Kp-10
FSH (IU/L)	1.80 ± 0.42	1.46 ± 0.25*	1.69 ± 0.21^#^	1.73 ± 0.29^#^	1.68 ± 0.19^#^
LH (IU/L)	1.08 ± 0.31	0.80 ± 0.17*	0.95 ± 0.13^#^	1.05 ± 0.19^##^	0.96 ± 0.16^#^
*GnRH m*RNA	1.00 ± 0.13	0.71 ± 0.21**	0.87 ± 0.05^#^	0.95 ± 0.06^##^	0.90 ± 0.06^#^
PRL (ng/L)	54.95 ± 9.46	61.34 ± 8.76*	56.47 ± 6.03^#^	54.55 ± 6.37^##^	59.25 ± 12.17
TSH (μIU/ml)	1.26 ± 0.23	1.48 ± 0.43*	1.26 ± 0.21^#^	1.40 ± 0.52	1.37 ± 0.39
TRH (ng/L)	0.98 ± 0.30	1.29 ± 0.47*	1.02 ± 0.23^#^	1.25 ± 0.29	1.30 ± 0.41

*P < 0.05, **P < 0.01 vs. control; ^#^P < 0.05, ^##^P < 0.01 vs. 10-TCS mice. L-T4, Levothyroxine; Quin, quinpirole; Kp-10, kisspeptin-10.

## Results

### Exposure of Female Mice to TCS Prolongs Diestrus

Female mice (12 weeks old) were treated with the oral gavage of TCS at 1, 10 and 100 mg/kg/day for 50 days, hereafter referred to as 1-TCS, 10-TCS, or 100-TCS mice (Figure 1). In comparison with controls (0.95 ± 0.28 ng/ml), the urinary TCS levels were increased by approximately 4-fold in 1-TCS mice (3.82 ± 1.59 ng/ml), 20-fold in 10-TCS mice (21.09 ± 5.78 ng/ml) and 40-fold in 100-TCS mice (40.82 ± 4.48 ng/ml), respectively. One-way repeated measures ANOVA revealed that TCS exposure did not affect body weight (*F*_(3,76)_ = 1.129, *P* > 0.05; Figure 2A): mean body weights in 1-TCS mice (37.40 ± 2.67 g), 10-TCS mice (37.69 ± 2.78 g) and 100-TCS mice (36.60 ± 2.49 g) were not significantly different than control mice (37.81 ± 2.24).

The estrous cycle was monitored using the vaginal smear test. As shown in Figure 2B, mice with regular 4–5 day cycles consisting of 1 day in proestrus followed by 1 day in estrus and 2–3 days in diestrus (including 1 day of metestrus) were called “regular cyclers”. Estrous cycle length (*F*_(3,76)_ = 18.31, *P* < 0.01; Figure 2C), and specifically diestrus length (*F*_(3,76)_ = 24.17, *P* < 0.01; Figure 2D) was altered by the TCS exposure. Approximately 65% of 10-TCS mice (*P* < 0.01) and 90% of 100-TCS mice (*P* < 0.01) had a persistent diestrus (lasting more than 5 days) starting from the 2nd week of TCS exposure.

### TCS Exposure Reduces Follicular Development and Ovulation

Ovary weights of 1-TCS mice (7.48 ± 1.55 mg), 10-TCS mice (7.69 ± 2.18 mg) and 100-TCS-mice (7.92 ± 1.44 mg) were not significantly different than control mice (8.12 ± 1.40 mg, *P* > 0.05, *n* = 20). To examine the possible influence of TCS on follicle development and ovulation, we counted the number of antral follicles and corpora lutea at diestrus using morphological criteria. As shown in Figure 3A, ovaries from control mice appeared typical for the diestrus stage. Numerous corpora lutea were observed in these ovaries, some showing clear evidence of recent ovulation. The numbers of antral follicles (*F*_(3,36)_ = 5.675, *P* < 0.05; Figure 3A-i) and corpora lutea (*F*_(3,36)_ = 3.371, *P* < 0.05; Figure 3A-ii) were affected by the TCS exposure, where the 10-TCS mice and 100-TCS mice showed a significant reduction in the number of antral follicles (*P* < 0.05) and corpora lutea (*P* < 0.05).

### TCS Exposure Causes Decline of HPG Axis

To explore the underlying mechanisms of TCS-caused persistent diestrus and ovary dysfunction, we measured the levels of reproductive hormones (Figure 1). At diestrus, the levels of serum FSH (*F*_(3,36)_ = 3.349, *P* < 0.05; Table 1), LH (*F*_(3,36)_ = 2.889, *P* < 0.05), P4 (*F*_(3,36)_ = 4.035, *P* < 0.05) and PRL (*F*_(3,76)_ = 3.376, *P* < 0.05) and *GnRH* mRNA (*F*_(3,36)_ = 9.422, *P* < 0.01), were all affected by TCS exposure. Specifically, 10-TCS mice and 100-TCS mice showed a modest but significant decrease in the levels of LH (*P* < 0.05 and *P* < 0.01), FSH (*P* < 0.05), P4 (*P* < 0.05) and *GnRH* mRNA (*P* < 0.01) compared to controls, which were associated with an obvious increase in the level of PRL (*P* < 0.05). Although the TCS exposure had a tendency to decrease the serum E2 level, this difference did not reach statistical significance (*F*_(3,36)_ = 2.083, *P* > 0.05). A surge-like LH release (LH surge) was observed between 1600 and 1700 in proestrus control mice and 1-TCS mice, but not in 10-TCS mice and 100-TCS mice (Figure 4A).

### TCS Exposure Suppresses Hypothalamic Kisspeptin Expression

To further investigate the targets of TCS-reduced HPG axis and LH surge production, we examined kisspeptin expression in AVPV of proestrus mice and ARC of diestrus mice. In proestrus control mice, the kisspeptin+ cells in AVPV (AVPV-kisspeptin+) were located along the third ventricle (Figure 4B). In comparison with control mice, the immunoreactivity of AVPV-kisspeptin+ cells in 10-TCS mice and 100-TCS mice was significantly reduced. In diestrus control mice, a large number of kisspeptin+ cells were observed in ARC (ARC-kisspeptin+ cells, Figure 4E). The immunoreactivity of ARC-kisspeptin+ cells in diestrus 10-TCS mice and 100-TCS mice was lower than that in control mice. In addition, either the level of AVPV-*kiss1* mRNA at proestrus (*F*_(3,36)_ = 11.512, *P* < 0.01; Figure 4C) or the level of ARC-*kiss1* mRNA at diestrus (*F*_(3,36)_ = 3.614, *P* < 0.05; Figure 4F) in 10-TCS mice and 100-TCS mice were significantly reduced. To avoid the influence of gonadal hormones and estrous cycles in kisspeptin expression, mice were OVX mice and then treated with E2 (100 μg/kg) to produce a preovulatory high level of E2 (Murphy, 2005). As shown in Figure 4D, the administration of E2 in control mice exerted a positive feedback regulation in the AVPV-kisspeptin expression (*P* < 0.01, *n* = 10). In comparison with OVX control mice, the level of AVPV-*kiss1* mRNA in OVX 10-TCS mice was reduced by approximately 40% (*P* < 0.01, *n* = 10). Although the high dose of E2 could elevate the level of AVPV-*kiss1* mRNA in OVX 10-TCS mice (*P* < 0.01, *n* = 10), their level of AVPV-*kiss1* mRNA still was less than half of E2-treated control mice (*P* < 0.01, *n* = 10).

### TCS-reduced Thyroid Hormones Leads to Hyperprolactinemia

To further explore the underlying mechanisms of TCS-enhanced secretion of PRL, we measured serum total thyroid hormones (T3 and T4), TSH and TRH (Figure 1). As shown in Table 1; the levels of T3 (*F*_(3,76)_ = 5.170, *P* < 0.01) and T4 (*F*_(3,76)_ = 15.267, *P* < 0.01), TSH (*F*_(3,76)_ = 3.919, *P* < 0.05) and TRH (*F*_(3,76)_ = 3.882, *P* < 0.05) were each affected by TCS exposure. While T3 and T4 were substantially reduced in 10-TCS (*P* < 0.05) and 100-TCS (*P* < 0.01) mice, TSH (*P* < 0.05) and TRH (*P* < 0.05) were increased. Interestingly, the administration of L-T4 at the dose of 20 μg/kg for 20 days starting from day 30 of 10-TCS exposure (Figure 1) reduced the previously observed increases in the levels of TSH (*P* < 0.05, *n* = 10; Table 2), TRH (*P* < 0.05, *n* = 10) and PRL (*P* < 0.05, *n* = 10).

### TCS-induced Hyperprolactinemia Suppresses Kisspeptin-Reproductive Endocrine

Thyroid hormone replacement in TCS mice corrected their hyperprolactinemia, next experiments were designed to examine whether the hyperprolactinemia causes the suppression of kisspeptin expression and hypothalamic-pituitary-reproductive endocrine. The results showed that the treatment of 10-TCS mice with L-T4 for 20 days was able to correct the decrease in the level of AVPV-*kiss1* mRNA at proestrus (*P* < 0.05, *n* = 10; Figure 5A) or ARC-*kiss1* mRNA at diestrus (*P* < 0.05, *n* = 10; Figure 5B), which was accompanied by a recovery of *GnRH* mRNA (*P* < 0.05, *n* = 10; Table 2), serum LH (*P* < 0.05, *n* = 10) and FSH (*P* < 0.05, *n* = 10).

Until now, there have been no commercially available the PRL receptor antagonists (Lan et al., 2017). To further determine the involvement of TCS-induced hyperprolactinemia in the down-regulation of kisspeptin-reproductive endocrine, we used a type-2 dopamine receptors agonist quinpirole (Quin), because a recent study (Nakano et al., 2010) has reported that TRH-induced PRL release is inhibited by the activation of type-2 dopamine receptors. As expected, the administration of quinpirole (2 mg/kg) for 20 days in 10-TCS mice could prevent the increase in the level of serum PRL (*P* < 0.05, *n* = 10; Table 2), but it had no effects on the elevation of TSH (*P* > 0.05, *n* = 10) and TRH (*P* > 0.05, *n* = 10). Similarly, the administration of quinpirole for 20 days in 10-TCS mice restored the levels of AVPV-*kiss1* mRNA (*P* < 0.05, *n* = 10), ARC-*kiss1* mRNA (*P* < 0.05, *n* = 10) and *GnRH* mRNA (*P* < 0.01, *n* = 10), as well as serum LH (*P* < 0.01, *n* = 10; Table 2) and FSH (*P* < 0.05, *n* = 10).

To demonstrate that TCS-induced hyperprolactinemia through reducing kisspeptin expression suppresses the reproductive endocrine, the 10-TCS mice were given the injection (i.c.v.) of the GPR45 agonist kisspeptin-10 (Kp-10) for 7 days (Figure 1). The results showed that the application of kisspeptin-10 was able to correct the decline of *GnRH* mRNA (*P* < 0.05, *n* = 10; Table 2), LH (*P* < 0.05, *n* = 10) and FSH (*P* < 0.05, *n* = 10) without altering the increased levels of TSH (*P* > 0.05, *n* = 10) and TRH (*P* > 0.05, *n* = 10), or PRL (*P* > 0.05, *n* = 10).

### TCS-suppressed Kisspeptin Impairs Estrous Cycle and Ovary Function

To confirm whether the TCS-suppressed kisspeptin neurons caused persistent diestrus and ovary dysfunction, 10-TCS mice were treated with L-T4 or quinpirole for 20 days after examined persistent diestrus (Figure 1). The results showed that treatment with L-T4 or quinpirole could return the diestrus lengths to normal (*P* < 0.05 and *P* < 0.01, *n* = 10; Figure 5C) and recover the numbers of antral follicles (*P* < 0.05, *n* = 10; Figure 5D) and corpora lutea (*P* < 0.05, *n* = 10; Figure 5E).

## Discussion

The results in the present study provides *in vivo* evidence that the exposure of adult female mice to TCS (≥10 mg/kg) reduces thyroid hormones, causing hyperprolactinemia which then suppresses hypothalamic kisspeptin neurons, ultimately disrupting reproductive endocrine and function.

Consistent with the TCS-reduced thyroid hormones in pregnant mice (Cao et al., 2017), exposing adult female mice to 10 and 100 mg/kg TCS caused the decline of T4 and T3. Wu et al. (2016) reported that exposure to TCS for such a short time (1 h) concentration-dependently decreases the sodium/iodide symporter (NIS)-mediated iodide uptake, which gave an inhibition constant (Ki) of 21.3 μM. TCS also inhibits thyroid peroxidase (TPO) activity (Wu et al., 2016). TPO catalyzes the oxidation of iodide and the addition of iodide to the tyrosine residues of thyroglobulin, making it critical to the synthesis of thyroid hormones (Taurog et al., 1996). In addition, TCS likely induces hypothyroxinemia in part through upregulation of hepatic catabolism, drastically increasing pentoxyresorufin O-dealkylase (PROD) activity (Paul et al., 2010). It has been reported that patients with hypothyroidism often show hyperprolactinemia (Watanobe and Sasaki, 1995). Reduced thyroid hormones in rats can lead to a significant increase in PRL level (Hapon et al., 2010). The decline of thyroid hormone probably enhances TRH secretion *via* reduced negative feedback of the hypothalamus-pituitary axis, which promotes the release of both TSH and PRL. The present data suggest that TCS-induced hypothyroidism presents the same scenario, as the elevated levels of serum PRL, TSH and TRH in 10-TCS mice were corrected by L-T4 administration. Hypothyroidism can also induce elevation in vasoactive intestinal peptide, which is capable of increasing PRL secretion (Tohei et al., 2000). On the other hand, PRL can regulate its own release by acting on the hypothalamic dopaminergic systems *via* a “short loop feedback”, which is mostly responsible for the maintenance of PRL homeostasis. Dopamine has been clearly established as the primary inhibitor of PRL release (Ben-Jonathan and Hnasko, 2001). The activation of type-2 dopamine receptors inhibits PRL release (Nakano et al., 2010) through rapidly increasing intracellular potassium efflux and reducing calcium influx (Lledo et al., 1990). The acute decline of serum PRL levels was observed in OVX rats treated with competitive dopamine transporter inhibitors (Demaria et al., 2000). Although we in this study did not examine the hypothalamic dopaminergic systems, we observed that the activation of type-2 dopamine receptors by quinpirole could reduce the level of circulating PRL in TCS mice without the changes in the levels of TRL and TSH. While Szawka et al. (2010) reported that injection (i.c.v.) of kisspeptin-10 to females elicits PRL release. However, in TCS mice the administration of kisspeptin-10 failed to alter the levels of PRL, TSH and TRH.

In female rats, a high proportion of ARC- and AVPV-kisspeptin neurons express the PRL receptors (Kokay et al., 2011). In OVX rats, the administration of PRL decreases the ARC- and AVPV-kisspeptin expression, and level of plasma LH (Araujo-Lopes et al., 2014). Acute administration of PRL for 1 h can also reduce the level of AVPV-*kiss1* mRNA (Higo et al., 2015). In TCS mice, the reduced ARC- or AVPV-kisspeptin expression was recovered by L-T4 or quinpirole that corrected the increase of PRL. Phosphorylation of signal transducer and activator of transcription 5 (STAT5) is known to be a reliable marker of PRL-responsive neurons (Brown et al., 2014). Exogenous PRL enhances phosphorylated STAT5 in kisspeptin neurons, while reduction of endogenous PRL inhibits STAT5 phosphorylation (Brown et al., 2014). PRL receptor and E2 receptor α (ERα) share a similar expression pattern in the ARC- and AVPV-kisspeptin neurons (Brown et al., 2011). The activation of ERα decreases the PRL-induced STAT5 transcriptional activity (Faulds et al., 2001). In TCS mice, the enhancing effect of E2 on the AVPV-kisspeptin expression was suppressed, which could be rescued by the quinpirole-corrected increase of PRL. Thus, it is proposed that the activation of PRL receptor suppresses E2-increased AVPV-kisspeptin expression. However, the administration of E2 in adult female mice negatively regulates the ARC-kisspeptin expression (Dungan et al., 2006). Moreover, the quinpirole-corrected increase of PRL could prevent the decline of ARC-kisspeptin expression in TCS mice. Thus, the suppression of kisspeptin synthesis by hyperprolactinemia is unlikely to act on ER-mediated signaling. The molecular mechanisms underlying the PRL-inhibited ARC- and AVPV-kisspeptin expression in TCS mice remains to be determined.

ARC- and AVPV-kisspeptin neurons exert critical afferent regulation in the activity of GnRH neurons (Pinilla et al., 2012). An abundance of evidence indicates that AVPV-kisspeptin neurons regulate the generation of GnRH and LH surge, and ARC-kisspeptin neurons are involved in the rhythm of GnRH pulse (Ohkura et al., 2009). Previous studies have identified that reduced ARC-*kiss1* mRNA and ARC-kisspeptin protein in lactating rats are associated with the suppression of pulsatile GnRH secretion (True et al., 2011); the administration of PRL prevents the occurrence of preovulatory LH surges in ovary-intact rats (Araujo-Lopes et al., 2014). We found no evidence of an LH surge in TCS mice unless they were also treated with L-T4 (data not shown). Hyperprolactinemia also causes anovulation in women by inhibiting the LH surge (McNeilly, 2001). In lactating mice, kisspeptin expression is reduced in both the AVPV and ARC, and lactational anovulation probably results from the selective loss of kisspeptin input to GnRH neurons (Liu et al., 2014). Here the decreases in the serum LH, FSH and P4 in TCS mice were corrected by either the quinpirole-corrected increase of PRL or the administration of kisspeptin-10. Kisspeptin replacement can also rescue fertility in hyperprolactinemic rats (Sonigo et al., 2012). In mammals, the lactation produces the suppression of GnRH/LH secretion, resulting in transient infertility. In TCS mice, the deficits in follicle development and ovulation could be rescued by L-T4 or the quinpirole-corrected increase of PRL, which was accompanied with regular estrous cycle. Thus, it is conceivable that TCS-induced hyperprolactinemia *via* the suppression of kisspeptin expression may impair the follicle development and ovulation. The level of E2 had no significant difference between control mice and TCS mice. One possible explanation is that the TCS exposure causes the inhibition of estrogen sulfotransferase (EST) to reduce the estradiol metabolism and clearance (James et al., 2010).

Based on urinary TCS levels from the National Health and Nutrition Examination Survey (NHANES) survey of 2003 and 2004 (range: 2.4–3, 790 μg/L; Calafat et al., 2008), mean daily intake has been estimated as low as 0.2–0.3 μg/kg/day, or up to about 47–73 μg/kg/day based on combined consumer product use (Rodricks et al., 2010). The most up to date NHANES survey of 2011–2012 suggests a similar range of urinary TCS levels (1.63–3, 830 μg/L; Witorsch, 2014). Wang et al. (2015) recently reported that the levels of urinary TCS are higher in some patients who experienced spontaneous abortion (11.21 μg/L) than those in normal pregnant women (0.99 μg/L). The urinary TCS levels in the pregnant mice treated with 10 mg/kg/day TCS for 5 days is equivalent to those of spontaneous abortion patients with high exposure to TCS. In contrast, the level of urinary TCS (the range of 21.09 ± 5.78 μg/L) in adult female mice exposed to the same dose of TCS for 50 days was increased nearly 2-fold. The plasma concentration of TCS in humans was increased rapidly after a single dose oral administration, attaining peak levels within 1–3 h, resulting in a terminal plasma half-life of 21 h (Sandborgh-Englund et al., 2006). However, laboratory experiments under aerobic conditions showed that TCS had a half-life of 18 days (Ying et al., 2007). Thus, one possible explanation is that the increase in urinary TCS level of 10-TCS mice may arise from an earlier sampling (12 h urinary after administration of TCS). In addition, further experiments should be done to clarify whether the chronic exposure to TCS affects the degradation rates of TCS leading to the accumulation of TCS. The amount of TCS that entered systemic circulation over the 24 h period is 12% in the feces, 1% in the urine, 30% in the stratum corneum, with 26% remaining on the surface of the skin (Moss et al., 2000). Measuring the level of TCS in urine, as an important biomonitoring tool, is used to reflect exposure assessment (Queckenberg et al., 2010). The primary elimination route of TCS is through fecal matter and urinary excretion, thus the levels of urinary TCS between 1-TCS mice, 10-TCS mice and 100-TCS mice did not show linear increase. Although the mechanisms underlying TCS-induced suppression of kisspeptin expression remain to be fully elucidated, our data in the present study indicate that exposure to TCS (≥10 mg/kg) in adult female mice through reducing thyroid hormones elevates the level of PRL, which can suppress the neuroendocrine reproductive axis, and ultimately leads to deficits in function of fertility. The findings can help for understanding the influence of TCS exposure in reproductive endocrine and reproductive health in humans.

## Author Contributions

X-YC, XH, and J-WX performed the experiment. X-YC and W-TZ analyzed the experimental data and wrote the manuscript. LC and JZ designed the studies and revised the manuscript. All authors approved the final version for publication.

## Conflict of Interest Statement

The authors declare that the research was conducted in the absence of any commercial or financial relationships that could be construed as a potential conflict of interest.

## References

[B1] Adachi S. Yamada S. Takatsu Y. Matsui H. Kinoshita M. Takase K.. (2007). Involvement of anteroventral periventricular metastin/kisspeptin neurons in estrogen positive feedback action on luteinizing hormone release in female rats. J. Reprod. Dev. 53, 367–378. 10.1262/jrd.18146 17213691

[B2] Ahn K. C. Zhao B. Chen J. Cherednichenko G. Sanmarti E. Denison M. S.. (2008). *In vitro* biologic activities of the antimicrobials triclocarban, its analogs, and triclosan in bioassay screens: receptor-based bioassay screens. Environ. Health Perspect. 116, 1203–1210. 10.1289/ehp.11200 18795164 PMC2535623

[B3] Araujo-Lopes R. Crampton J. R. Aquino N. S. Miranda R. M. Kokay I. C. Reis A. M.. (2014). Prolactin regulates kisspeptin neurons in the arcuate nucleus to suppress LH secretion in female rats. Endocrinology 155, 1010–1020. 10.1210/en.2013-1889 24456164

[B4] Ben-Jonathan N. Hnasko R. (2001). Dopamine as a prolactin (PRL) inhibitor. Endocr. Rev. 22, 724–763. 10.1210/er.22.6.724 11739329

[B5] Brown R. S. Herbison A. E. Grattan D. R. (2011). Differential changes in responses of hypothalamic and brainstem neuronal populations to prolactin during lactation in the mouse. Biol. Reprod. 84, 826–836. 10.1095/biolreprod.110.089185 21178171

[B6] Brown R. S. Herbison A. E. Grattan D. R. (2014). Prolactin regulation of kisspeptin neurones in the mouse brain and its role in the lactation-induced suppression of kisspeptin expression. J. Neuroendocrinol. 26, 898–908. 10.1111/jne.12223 25207795

[B7] Calafat A. M. Ye X. Wong L. Y. Reidy J. A. Needham L. L. (2008). Urinary concentrations of triclosan in the U.S. population: 2003–2004. Environ. Health Perspect. 116, 303–307. 10.1289/ehp.10768 18335095 PMC2265044

[B8] Caligioni C. S. (2009). Assessing reproductive status/stages in mice. Curr. Protoc. Neurosci. Appendix 4:Appendix 4I. 10.1002/0471142301.nsa04is48 19575469 PMC2755182

[B9] Cao X. Hua X. Wang X. Chen L. (2017). Exposure of pregnant mice to triclosan impairs placental development and nutrient transport. Sci. Rep. 7:44803. 10.1038/srep44803 28322267 PMC5359620

[B10] Chalew T. E. Halden R. U. (2009). Environmental exposure of aquatic and terrestrial biota to triclosan and triclocarban. J. Am. Water Works Assoc. 45, 4–13. 10.1111/j.1752-1688.2008.00284.x 20046971 PMC2684649

[B11] Clarkson J. Han S. Y. Piet R. McLennan T. Kane G. M. Ng J.. (2017). Definition of the hypothalamic GnRH pulse generator in mice. Proc. Natl. Acad. Sci. U S A 114, E10216–E10223. 10.1073/pnas.1713897114 29109258 PMC5703322

[B12] Demaria J. E. Nagy G. M. Lerant A. A. Fekete M. I. Levenson C. W. Freeman M. E. (2000). Dopamine transporters participate in the physiological regulation of prolactin. Endocrinology 141, 366–374. 10.1210/en.141.1.366 10614659

[B13] Dungan H. M. Clifton D. K. Steiner R. A. (2006). Minireview: kisspeptin neurons as central processors in the regulation of gonadotropin-releasing hormone secretion. Endocrinology 147, 1154–1158. 10.1210/en.2005-1282 16373418

[B14] Faulds M. H. Pettersson K. Gustafsson J. A. Haldosén L. A. (2001). Cross-talk between ERs and signal transducer and activator of transcription 5 is E2 dependent and involves two functionally separate mechanisms. Mol. Endocrinol. 15, 1929–1940. 10.1210/me.15.11.1929 11682624

[B15] Goel P. Kahkasha Narang S. Gupta B. K. Goel K. (2015). Evaluation of serum prolactin level in patients of subclinical and overt hypothyroidism. J. Clin. Diagn. Res. 9, BC15–BC17. 10.7860/JCDR/2015/9982.5443 25737975 PMC4347066

[B16] Gottsch M. L. Cunningham M. J. Smith J. T. Popa S. M. Acohido B. V. Crowley W. F.. (2004). A role for kisspeptins in the regulation of gonadotropin secretion in the mouse. Endocrinology 145, 4073–4077. 10.1210/en.2004-0431 15217982

[B17] Hapon M. B. Gamarra-Luques C. Jahn G. A. (2010). Short term hypothyroidism affects ovarian function in the cycling rat. Reprod. Biol. Endocrinol. 8:14. 10.1186/1477-7827-8-14 20149258 PMC2841189

[B18] Higo S. Aikawa S. Iijima N. Ozawa H. (2015). Rapid modulation of hypothalamic Kiss1 levels by the suckling stimulus in the lactating rat. J. Endocrinol. 227, 105–115. 10.1530/JOE-15-0143 26446276

[B19] James M. O. Li W. Summerlot D. P. Rowland-Faux L. Wood C. E. (2010). Triclosan is a potent inhibitor of estradiol and estrone sulfonation in sheep placenta. Environ. Int. 36, 942–949. 10.1016/j.envint.2009.02.004 19299018 PMC4789100

[B20] Jung E. M. An B. S. Choi K. C. Jeung E. B. (2012). Potential estrogenic activity of triclosan in the uterus of immature rats and rat pituitary GH3 cells. Toxicol. Lett. 208, 142–148. 10.1016/j.toxlet.2011.10.017 22062131

[B21] Kinoshita M. Tsukamura H. Adachi S. Matsui H. Uenoyama Y. Iwata K.. (2005). Involvement of central metastin in the regulation of preovulatory luteinizing hormone surge and estrous cyclicity in female rats. Endocrinology 146, 4431–4436. 10.1210/en.2005-0195 15976058

[B22] Kokay I. C. Petersen S. L. Grattan D. R. (2011). Identification of prolactin-sensitive GABA and kisspeptin neurons in regions of the rat hypothalamus involved in the control of fertility. Endocrinology 152, 526–535. 10.1210/en.2010-0668 21177834

[B23] Lan H. Hong P. Li R. L S. Anshan S. Li S.. (2017). Internal image anti-idiotypic antibody: a new strategy for the development a new category of prolactin receptor (PRLR) antagonist. Mol. Immunol. 87, 86–93. 10.1016/j.molimm.2017.04.006 28412548

[B24] Liu X. Brown R. S. Herbison A. E. Grattan D. R. (2014). Lactational anovulation in mice results from a selective loss of kisspeptin input to GnRH neurons. Endocrinology 155, 193–203. 10.1210/en.2013-1621 24169550

[B25] Lledo P. M. Legendre P. Zhang J. Israel J. M. Vincent J. D. (1990). Effects of dopamine on voltage-dependent potassium currents in identified rat lactotroph cells. Neuroendocrinology 52, 545–555. 10.1159/000125650 2149427

[B26] Louis G. W. Hallinger D. R. Stoker T. E. (2013). The effect of triclosan on the uterotrophic response to extended doses of ethinyl estradiol in the weanling rat. Reprod. Toxicol. 36, 71–77. 10.1016/j.reprotox.2012.12.001 23261820

[B27] Marraudino M. Miceli D. Farinetti A. Ponti G. Panzica G. Gotti S. (2017). Kisspeptin innervation of the hypothalamic paraventricular nucleus: sexual dimorphism and effect of estrous cycle in female mice. J. Anat. 230, 775–786. 10.1111/joa.12603 28295274 PMC5442148

[B28] Mayer C. Boehm U. (2011). Female reproductive maturation in the absence of kisspeptin/GPR54 signaling. Nat. Neurosci. 14, 704–710. 10.1038/nn.2818 21516099

[B29] McNeilly A. S. (2001). Lactational control of reproduction. Reprod. Fertil. Dev. 13, 583–590. 10.1071/RD01056 11999309

[B30] Moss T. Howes D. Williams F. M. (2000). Percutaneous penetration and dermal metabolism of triclosan (2,4, 4′-trichloro-2′-hydroxydiphenyl ether). Food Chem. Toxicol. 38, 361–370. 10.1016/s0278-6915(99)00164-7 10722890

[B31] Murphy K. G. (2005). Kisspeptins: regulators of metastasis and the hypothalamic-pituitary-gonadal axis. J. Neuroendocrinol. 17, 519–525. 10.1111/j.1365-2826.2005.01328.x 16011488

[B32] Myers M. Britt K. L. Wreford N. G. Ebling F. J. Kerr J. B. (2004). Methods for quantifying follicular numbers within the mouse ovary. Reproduction 127, 569–580. 10.1530/rep.1.00095 15129012

[B33] Nakano M. Minagawa A. Hasunuma I. Okada R. Tonon M. C. Vaudry H.. (2010). D2 Dopamine receptor subtype mediates the inhibitory effect of dopamine on TRH-induced prolactin release from the bullfrog pituitary. Gen. Comp. Endocrinol. 168, 287–292. 10.1016/j.ygcen.2010.05.008 20553721

[B34] Navarro V. M. Castellano J. M. Fernández-Fernández R. Barreiro M. L. Roa J. Sanchez-Criado J. E.. (2004). Developmental and hormonally regulated messenger ribonucleic acid expression of KiSS-1 and its putative receptor, GPR54, in rat hypothalamus and potent luteinizing hormone-releasing activity of KiSS-1 peptide. Endocrinology 145, 4565–4574. 10.1210/en.2004-0413 15242985

[B35] Ohkura S. Uenoyama Y. Yamada S. Homma T. Takase K. Inoue N.. (2009). Physiological role of metastin/kisspeptin in regulating gonadotropin-releasing hormone (GnRH) secretion in female rats. Peptides 30, 49–56. 10.1016/j.peptides.2008.08.004 18775461

[B36] Paul K. B. Hedge J. M. DeVito M. J. Crofton K. M. (2010). Short-term exposure to triclosan decreases thyroxine *in vivo* via upregulation of hepatic catabolism in young Long-Evans rats. Toxicol. Sci. 113, 367–379. 10.1093/toxsci/kfp271 19910387 PMC2902919

[B37] Pinilla L. Aguilar E. Dieguez C. Millar R. P. Tena-Sempere M. (2012). Kisspeptins and reproduction: physiological roles and regulatory mechanisms. Physiol. Rev. 92, 1235–1316. 10.1152/physrev.00037.2010 22811428

[B38] Pycke B. F. Geer L. A. Dalloul M. Abulafia O. Jenck A. M. Halden R. U. (2014). Human fetal exposure to triclosan and triclocarban in an urban population from Brooklyn, New York. Environ. Sci. Technol. 48, 8831–8838. 10.1021/es501100w 24971846 PMC4123932

[B39] Qiu J. Nestor C. C. Zhang C. Padilla S. L. Palmiter R. D. Kelly M. J.. (2016). High-frequency stimulation-induced peptide release synchronizes arcuate kisspeptin neurons and excites GnRH neurons. Elife 5:e16246. 10.7554/eLife.16246 27549338 PMC4995096

[B40] Queckenberg C. Meins J. Wachall B. Doroshyenko O. Tomalik-Scharte D. Bastian B.. (2010). Absorption, pharmacokinetics, and safety of triclosan after dermal administration. Antimicrob. Agents Chemother. 54, 570–572. 10.1128/aac.00615-09 19822703 PMC2798550

[B41] Reiss R. Lewis G. Griffin J. (2009). An ecological risk assessment for triclosan in the terrestrial environment. Environ. Toxicol. Chem. 28, 1546–1556. 10.1897/08-250.1 19228078

[B42] Rodricks J. V. Swenberg J. A. Borzelleca J. F. Maronpot R. R. Shipp A. M. (2010). Triclosan: a critical review of the experimental data and development of margins of safety for consumer products. Crit. Rev. Toxicol. 40, 422–484. 10.3109/10408441003667514 20377306

[B43] Rodríguez P. E. A. Sanchez M. S. (2010). Maternal exposure to triclosan impairs thyroid homeostasis and female pubertal development in Wistar rat offspring. J. Toxicol. Environ. Health A 73, 1678–1688. 10.1080/15287394.2010.516241 21058171

[B44] Sandborgh-Englund G. Adolfsson-Erici M. Odham G. Ekstrand J. (2006). Pharmacokinetics of triclosan following oral ingestion in humans. J. Toxicol. Environ. Health Part A 69, 1861–1873. 10.1080/15287390600631706 16952905

[B45] Sonigo C. Bouilly J. Carré N. Tolle V. Caraty A. Tello J.. (2012). Hyperprolactinemia-induced ovarian acyclicity is reversed by kisspeptin administration. J. Clin. Invest. 122, 3791–3795. 10.1172/JCI63937 23006326 PMC3461919

[B46] Stathatos N. Bourdeau I. Espinosa A. V. Saji M. Vasko V. V. Burman K. D.. (2005). KiSS-1/G protein-coupled receptor 54 metastasis suppressor pathway increases myocyte-enriched calcineurin interacting protein 1 expression and chronically inhibits calcineurin activity. J. Clin. Endocrinol. Metab. 90, 5432–5440. 10.1210/jc.2005-0963 15998767

[B47] Stoker T. E. Gibson E. K. Zorrilla L. M. (2010). Triclosan exposure modulates estrogen-dependent responses in the female wistar rat. Toxicol. Sci. 117, 45–53. 10.1093/toxsci/kfq180 20562219

[B48] Szawka R. E. Ribeiro A. B. Leite C. M. Helena C. V. Franci C. R. Anderson G. M.. (2010). Kisspeptin regulates prolactin release through hypothalamic dopaminergic neurons. Endocrinology 151, 3247–3257. 10.1210/en.2009-1414 20410200

[B49] Taurog A. Dorris M. L. Doerge D. R. (1996). Mechanism of simultaneous iodination and coupling catalyzed by thyroid peroxidase. Arch. Biochem. Biophys. 330, 24–32. 10.1006/abbi.1996.0222 8651700

[B50] Tohei A. Taya K. Watanabe G. Voogt J. L. (2000). Hypothyroidism increases prolactin secretion and decreases the intromission threshold for induction of pseudopregnancy in adult female rats. Physiol. Behav. 69, 391–397. 10.1016/s0031-9384(00)00224-9 10913776

[B51] True C. Kirigiti M. Ciofi P. Grove K. L. Smith M. S. (2011). Characterisation of arcuate nucleus kisspeptin/neurokinin B neuronal projections and regulation during lactation in the rat. J. Neuroendocrinol. 23, 52–64. 10.1111/j.1365-2826.2010.02076.x 21029216 PMC3118985

[B53] Wang X. Chang F. Bai Y. Chen F. Zhang J. Chen L. (2014). Bisphenol A enhances kisspeptin neurons in anteroventral periventricular nucleus of female mice. J. Endocrinol. 221, 201–213. 10.1530/joe-13-0475 24532816

[B54] Wang X. Chen X. Feng X. Chang F. Chen M. Xia Y.. (2015). Triclosan causes spontaneous abortion accompanied by decline of estrogen sulfotransferase activity in humans and mice. Sci. Rep. 5:18252. 10.1038/srep18252 26666354 PMC4678904

[B52] Wang X. Ouyang F. Feng L. Wang X. Liu Z. Zhang J. (2017). Maternal urinary triclosan concentration in relation to maternal and neonatal thyroid hormone levels: a prospective study. Environ. Health Perspect. 125:067017. 10.1289/ehp500 28669941 PMC5743753

[B55] Watanobe H. Sasaki S. (1995). Effect of thyroid status on the prolactin-releasing action of vasoactive intestinal peptide in humans: comparison with the action of thyrotropin-releasing hormone. Neuroendocrinology 61, 207–212. 10.1159/000126842 7753340

[B56] Witorsch R. J. (2014). Critical analysis of endocrine disruptive activity of triclosan and its relevance to human exposure through the use of personal care products. Crit. Rev. Toxicol. 44, 535–555. 10.3109/10408444.2014.910754 24897554

[B57] Wu Y. Beland F. A. Fang J. L. (2016). Effect of triclosan, triclocarban, 2,2′,4,4′-tetrabromodiphenyl ether, and bisphenol A on the iodide uptake, thyroid peroxidase activity, and expression of genes involved in thyroid hormone synthesis. Toxicol. In Vitro 32, 310–319. 10.1016/j.tiv.2016.01.014 26827900

[B58] Xi W. Lee C. K. Yeung W. S. Giesy J. P. Wong M. H. Zhang X.. (2011). Effect of perinatal and postnatal bisphenol A exposure to the regulatory circuits at the hypothalamus-pituitary-gonadal axis of CD-1 mice. Reprod. Toxicol. 31, 409–417. 10.1016/j.reprotox.2010.12.002 21182934

[B59] Ying G. G. Yu X. Y. Kookana R. S. (2007). Biological degradation of triclocarban and triclosan in a soil under aerobic and anaerobic conditions and comparison with environmental fate modelling. Environ. Pollut. 150, 300–305. 10.1016/j.envpol.2007.02.013 17459543

[B60] Zhang T. Hong J. Di T. Chen L. (2016). MPTP impairs Dopamine D1 receptor-mediated survival of newborn neurons in ventral hippocampus to cause depressive-like behaviors in adult mice. Front. Mol. Neurosci. 9:101. 10.3389/fnmol.2016.00101 27790091 PMC5062058

